# Developing a State University System Model to Diversify Faculty in the Biomedical Sciences

**DOI:** 10.3389/fpsyg.2022.734145

**Published:** 2022-03-17

**Authors:** Robin Herlands Cresiski, Cynthia Anne Ghent, Janet C. Rutledge, Wendy Y. Carter-Veale, Jennifer Aumiller, John Carlo Bertot, Blessing Enekwe, Erin Golembewski, Yarazeth Medina, Michael S. Scott

**Affiliations:** ^1^University of Maryland Baltimore County, Baltimore, MD, United States; ^2^Towson University, Towson, MD, United States; ^3^University of Maryland Baltimore, Baltimore, MD, United States; ^4^University of Maryland College Park, College Park, MD, United States; ^5^Salisbury University, Salisbury, MD, United States

**Keywords:** faculty diversity, biomedical sciences, postdoc, AGEP, state university system

## Abstract

Amid increasing demands from students and the public, universities have recently reinvigorated their efforts to increase the number of faculty from underrepresented populations. Although a myriad of piecemeal programs targeting individual recruitment and development have been piloted at several institutions, overall growth in faculty diversity remains almost negligible and highly localized. To bring about genuine change, we hypothesize a consortia approach that links individuals to hiring opportunities within a state university system might be more effective. Here we present a case study describing the progress of the NSF-funded Alliances for Graduate Education and the Professoriate (AGEP) PROMISE Academy Alliance, a consortium within the University System of Maryland (USM) collaborating to develop, implement, self-study, evaluate, and disseminate a unique postdoc-to-faculty conversion model in the biomedical sciences. The initiative centers on diversifying faculty across five institutions in the USM, including teaching-focused institutions, comprehensive universities, research institutions, and professional schools. Components of this approach include (1) enhanced recruiting and hiring practices to attract outstanding postdoctoral scholars from underrepresented backgrounds, (2) multi-institutional networking and professional development, and (3) facilitated processes to transition (or “convert”) postdocs into tenure-track positions at their postdoctoral institution or another institution in the state system. This model is distinct from more deficit-based approaches because it goes beyond focusing on building the individual’s skills to enter the professoriate. This program restructures the traditionally short-term nature of postdoctoral employment and incorporates a pathway to a tenure-track professorship at the same institution or within the same statewide system where the postdoc is trained. This multi-institutional model leverages collaboration and distinct institutional strengths to create cross-institutional support, advocacy, and policy. Importantly, it uses a decentralized financial structure that makes this approach distinctly replicable. Recognizing the immediate need for more collaborative approaches to diversify faculty and a lack of literature about such approaches, this case study describes the development of, and potential benefits of, a state university system, as well as the qualitative lessons learned from self-study, internal evaluation, external evaluation, and NSF site visits. The AGEP PROMISE Academy can serve as a model for replication at other university systems hoping to diversify their faculty.

## Introduction

Increasing faculty diversity has important implications for maintaining and growing U.S. competitiveness in innovation, the knowledge and science economy, and broadly equipping the 21st-century workforce. The compelling need for more innovative approaches to diversify faculty is clear by the changing demographics of the student body and the ensuing racial and ethnic imbalance. While the national percentage of underrepresented minority college students (undergraduate and graduate students combined) has risen to nearly 50%, the percentage of underrepresented minority faculty remains below 30% (Finkelstein et al., 2016; Snyder et al., 2018; Espinosa et al., 2019; Brown, 2021), and the percentage of tenure-track minority faculty remains even lower at 22% (NCES, 2018). The benefits of a more diverse faculty extend to all students (Stout et al., 2018). For students from traditionally underrepresented racial and ethnic groups, having faculty role models from similar backgrounds sends a powerful message of support and belonging (Jayakumar et al., 2009; Cole and Griffin, 2013; Shin et al., 2016; Griffin, 2020), and students from majority backgrounds gain by experiencing broader pedagogical perspectives (Umbach, 2006) and countering stereotypes to reduce bias (Gocłowska and Crisp, 2013). For these reasons and many others, successful initiatives to increase the number of faculty from underrepresented backgrounds are critical.

The academy has been discussing strategies to improve racial equity for decades, but progress has been incremental and slow (Snyder et al., 2018). Unfortunately, there are still many structural barriers that impede excellent underrepresented STEM postdoctoral scholars from being recruited, retained, and promoted into faculty positions. Because postdoctoral training is the gateway to a tenure-track position in the biomedical sciences, the structural barriers to accessing employment at this level help to maintain the stark racial and ethnic disparities in outcomes at the faculty level. For example, lack of access to opportunities to learn about academic careers (Gibbs et al., 2015) or to obtain professional and social supports (Layton et al., 2016) as well as clearly documented racial bias in postdoctoral (Eaton et al., 2020) and faculty search processes (White-Lewis, 2020) prevent entry into faculty positions. Beyond recruitment and hiring, other structural barriers can include a toxic department culture (Cole and Hassel, 2017; Dutt-Ballerstadt, 2020), or a culture of “niceness” that centers on conflict avoidance (Liera, 2020), a disproportionate workload (Peek et al., 2013; Jimenez et al., 2019; O’Meara et al., 2019; Dutt-Ballerstadt, 2020; Flaherty, 2020) and lack of attention to the importance of sense of belonging (Gibbs et al., 2015) which contributes to the failure of institutions to retain scholars of color in the academy. When recruited and hired in low numbers, biomedical faculty from underrepresented backgrounds are often socially isolated and less likely to find connections of shared experiences (Misra et al., 2021) and thus find themselves with limited mental and emotional support when they most need it.

One potential avenue to securing more diverse faculty is to recruit more underrepresented postdoctoral scholars. In the laboratory sciences and a growing number of other disciplines, postdoctoral appointments (where scholars work on the research of a faculty member) are an expectation prior to securing a tenure-track position. These appointments are typically 2–3 years long, but a recent *Nature* survey of postdoctoral scholars found that 48% of respondents had been working as a postdoc for more than 3 years, with 30% of respondents having already completed two or three positions before their current postdoctoral appointment (Woolston, 2020a). Academic careers are the top choice of postdoctoral scholars, with over half of biomedical postdocs ranking faculty positions as their intended career, but interest in pursuing academia typically decreases between years one and three of a postdoc, particularly for underrepresented minorities and women (Lambert et al., 2020; Woolston, 2020b). There is justification for the noted pessimism and anxiety documented in postdoctoral surveys: only 15–20% of postdocs actually do transition to tenure-track positions (Kahn and Ginther, 2017; McConnell et al., 2018). The postdoc-to-faculty transition has been recognized as one of two key junctures where underrepresented minorities divert from their goals of becoming faculty (Meyers et al., 2018) and financial security, responsibility to family, and lower sense of belonging and self-efficacy seem highly influential in the departure of female and underrepresented scholars (Lambert et al., 2020). However, few interventions focus on this critical period or the barriers presented by traditional postdoctoral positions.

Institutional efforts have been tried. For example, prestigious, postdoctoral fellowships, designed specifically for scholars from underrepresented backgrounds, are growing in number, attempting to attract more scholars from the doctorate into pre-faculty roles. Despite documented successes of these programs (Holtzclaw et al., 2005; Faupel-Badger and Miklos, 2016; Eisen and Eaton, 2017) they do not address transitional barriers head on. A promising intervention is the postdoctoral conversion model, where scholars from underrepresented backgrounds are recruited into postdoctoral positions that come with a direct pathway to “convert” to the tenure-track at their fellowship institution (Culpepper et al., 2021). Because this reduces a barrier to the professoriate position and provides financial security, it stands a chance of making a significant difference in enhancing racial equity. Conversion models, however, are being implemented at only 38 institutions nationally (Culpepper et al., 2021) out of over 5,000 colleges and universities, making any potential progress slow and localized to individual departments or institutions.

Scaling up conversion models to the university system may be a way to accelerate their potential. Dr. Kimberly Griffin, author of *Redoubling Our Efforts: How Institutions Can Affect Faculty Diversity*, is “increasingly convinced that collaborative efforts were the key to real gains in faculty diversity across higher education… group efforts might happen not just across disciplines, with the help of disciplinary organizations, but also in other configurations—such as across a state university system” (Flaherty, 2016). While some university systems have strategic plans for increasing the number of faculty from underrepresented backgrounds, commitment statements, or even “action plans,” rarely accompany any tangible steps being taken to combat this problem meaningfully. In fact, the only pre-existing exemplar of a system-wide approach is the University of California system’s President’s Postdoctoral Fellowship Program, established in 1984 to “encourage outstanding minority Ph.D. recipients to pursue academic careers at the University of California.” Not only does it supply funded postdoctoral positions, in 2003, it began incentivizing the tenure-track hire of these scholars by providing 5 years of salary support and start-up funds. This program has been incredibly successful, with over 260 hires of minority scholars into tenure-track positions since the financial incentives were established (Lawson, 2020). However, no research has been published about the establishment, evolution, or efficacy of this model, and the centralized funding approach used by the University of California system (facilitating initiatives where funds can be dispensed to institutions within the system easily) is uncommon among state university systems, and thus has not been replicated.

On the other side of the country, within the University System of Maryland (USM), a new state system approach is being developed that could have greater scalability because it operates in the context of a more traditional university system, with institutional budgets set by the state and extremely limited funds for centralized initiatives. In this article, we describe the current progress of Maryland’s NSF-funded Alliances for Graduate Education and the Professoriate (AGEP) PROMISE Academy Alliance, a five-institution consortium (out of 12 institutions that comprise the USM) developing a model to increase the number of tenure-track underrepresented faculty in the biomedical sciences. Building on successful lessons of other postdoctoral programs aimed at supporting the success of underrepresented scholars, the AGEP PROMISE Academy Alliance seeks to recruit, onboard, develop, and mentor postdoctoral fellows to be prepared for the tenure-track. Uniquely, this program includes overt intention and concrete support to transition postdoctoral fellows into tenure-track faculty positions, either at their postdoctoral institution *or at another institution within the university system*. This disrupts the traditional short-term timeframe of a postdoc and hopefully some of the subsequent insecurity and anxiety that accompanies standard postdoctoral fellowships (Postdoctoral training: time for change, 2011; Milojević et al., 2018; Woolston, 2020a). The stress of this insecurity is especially daunting for scholars with children or those hoping to have children (De Welde and Laursen, 2011; Woolston, 2020a), putting women from underrepresented backgrounds at a particular intersectional disadvantage. The AGEP PROMISE Academy also provides fellows the benefit of networking and learning about different types of institutions, something frequently absent from a postdoctoral fellowship, and provides potential hiring institutions with a supply of highly qualified, vetted, and trained scholars as potential colleagues.

Below, we present a case study of this novel intervention, describing the key programmatic elements of the AGEP PROMISE Academy Alliance model along with qualitative data assembled from focus groups, document analysis, meeting observations and interviews, collected through self-study as well as internal evaluation, external evaluation, and multiple NSF site visits. This article summarizes many of the facilitators and hindrances observed and reported by evaluators to provide insight into the development of both a state system alliance and as well as a unique fellowship program for underrepresented postdocs. While data on the impact of the model is limited (due to being just 3 years into implementation), robust data has been collected about the *process* of developing this multi-level collaborative intervention. Considering the dearth of literature on system approaches to faculty diversity and the high interest of institutions and systems to make more substantive progress, we include discussion of barriers to developing state system alliances, successes that can be and have been measured during development, and practical lessons learned in our effort to increase the hiring and retention of faculty from underrepresented populations in five institutions within one university system.

## Context and Background

The AGEP Promise Academy Alliance has a focus on diversifying the faculty in biomedical sciences and includes five institutions within the USM: two research-intensive campuses [the University of Maryland College Park (UMCP), and the University of Maryland Baltimore County (UMBC)], two comprehensive teaching-focused universities (Salisbury University, SU, and Towson University, TU), and a research-intensive professional school (University of Maryland Baltimore, UMB). The research-intensive campuses and the professional school (UMCP, UMBC, and UMB) had a history of working together to provide support and programming for underrepresented graduate students in STEM through previous NSF AGEP awards, and that relationship served as a strong foundation on which to build a system-wide model geared at the next stage of the professoriate career path: the postdoctoral position. The alliance is building a model that uses enhanced recruiting and hiring practices to attract outstanding postdoctoral scholars (Fellows), provides a multi-institutional professional development plan (leveraging distinct strengths of institutions within the alliance), and creates facilitated conversion processes to transition postdocs into tenure-track positions at their postdoctoral institution or another institution in the statewide system. The alliance provides a unique development program (the AGEP PROMISE Academy) for Fellows by leveraging the strengths and differences of all partner institutions. The model includes two conversion pathways: (1) the Predetermined pathway supports a Fellow through the program with the expectation to convert the Fellow into a faculty position at the same institution where the postdoctoral fellowship is completed, and (2) the Flexible pathway supports a Fellow to investigate and connect with other institutions within the university system with aims of transitioning the Fellow into a tenure-track faculty position. The AGEP PROMISE Academy supports postdoctoral scholars as they prepare to enter tenure-track faculty positions after experiences with career- and skill-building professional development, dedicated mentoring and networking, and opportunities to showcase their research at other USM campuses. As all institutions had strong biomedical programs, including behavioral and cognitive sciences, the model places an emphasis on diversifying faculty in the biomedical sciences. The alliance is funded by the NSF and is in the beginning of year four of the five-year grant period.

The USM consists of 12 Institutions, three Centers, and one System Office, spread across the state, and serves over 170,000 students. Geographically, Maryland is small, with most university system institutions within easy driving distance from one another. While operating under the umbrella of the university system, each institution is autonomous, with separate presidents, provosts, and budgets. Collectively, these independent leaders unite to formulate common strategies and policies for the entire system. The unique individuality of institutions is a strength to our system and the inherent diversity this individuality brings allows for successful collaborations. Diversity and inclusion have been at the forefront of Maryland’s university system and on individual campuses. A recent example of this dedication was shown when UMCP, recently announced a $40 M investment to promote efforts to attract, hire, and support more faculty from diverse backgrounds. Many USM institutions have official diversity, equity, and inclusion offices and officers, all of which provide support and assistance for increasing and supporting diversity initiatives.

## A New Model for Faculty Diversification

### The State University System Approach: A Multi-Level Collaboration

The goal of Maryland’s AGEP PROMISE Academy Alliance is to develop, implement, self-study, evaluate, and disseminate (DISED) a state system alliance model to increase the number of tenure-track faculty from underrepresented (as defined by the National Science Foundation)^1^ backgrounds in the biomedical sciences within the system. The work of this project is three-pronged: (1) we are generating a postdoctoral program and experience (the AGEP PROMISE Academy) that includes recruitment, selection, mentorship, professional development, and conversion into tenure-track positions (at the fellowship institution or another institution with state university system); (2) we are creating, assessing and evolving the structures needed for a system-wide project to operate; and (3) simultaneously, an arm of our alliance is conducting significant research on bias in faculty search processes (creating the “R” in what the NSF refers to as DISED+R). This article focuses on the first two prongs of the project, as the research component is parallel to, and not an assessment of, the first two prongs. This work is shaped considerably around the five pillars of Collective Impact Strategy (CIS; Kania and Kramer, 2011): building a common vision, using agreed-upon metrics of evaluation, facilitating mutually reinforcing activities, encouraging continuous communication, and establishing a strong backbone of dedicated staff to ensure the sustainability of the project. These pillars will be referred to throughout this article.

**Figure 1:**
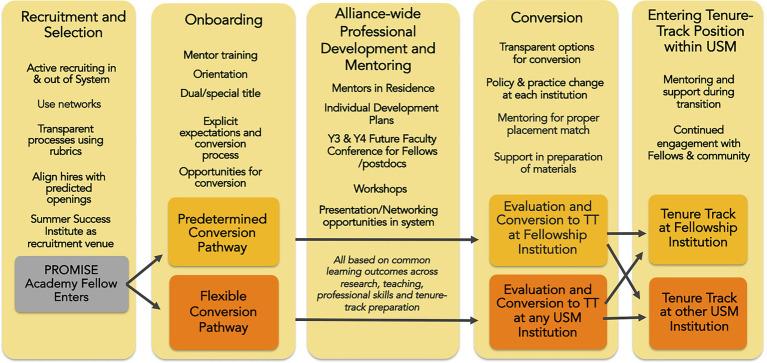
Components of the Alliances for Graduate Education and the Professoriate (AGEP) PROMISE Academy, a program to recruit, onboard, professionally develop, and convert biomedical postdocs from underrepresented backgrounds into tenure-track faculty positions, either at their fellowship institution (Predetermined pathway) or at another institution within the state university system (Flexible pathway).

**Figure 2:**
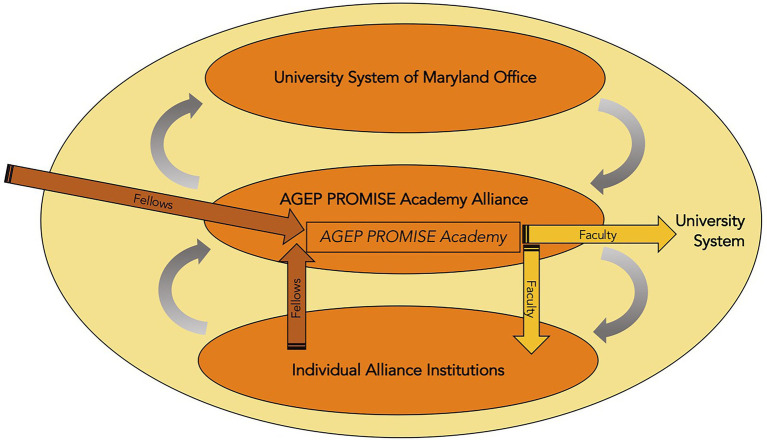
Conceptual model of interaction (gray arrows) between University System of Maryland’s System Office, AGEP PROMISE Academy Alliance, and the individual AGEP PROMISE Academy Alliance institutions. AGEP PROMISE Academy Fellows enter the program (rust arrows) from institutions within the Alliance, within the university system, or from external institutions and move into tenure track faculty positions (gold arrows) following the Predetermined pathway to a position at their home Alliance institution or the Flexible pathway to a position at a different Alliance institution or other USM institution.

The programmatic experience created for postdoctoral scholars from underrepresented backgrounds, the AGEP PROMISE Academy, was conceived from recruitment, onboarding, and professional development to conversion to a tenure-track position (see Figure 1). A broad team of over 35 individuals across alliance institutions (including provosts, deans, directors, staff, and faculty) collaborated to create this program at a kick-off retreat in the first year of the project. The details and merits of the programming will be discussed later in this section. Our model for diversification within a state university system centers the AGEP PROMISE Academy program, but also emphasizes the continuous interaction and influence between the Alliance, the individual institutions within the Alliance, and the university system office (see Figure 2). As an Alliance, we created the model and program communally and have together developed protocols, guidelines, and tools to facilitate implementation of the program across the Alliance. However, we continuously learn from and leverage institutional expertise, and also execute elements of our model and program through complementary institutional processes (Figure 2). For example, our model has been informed heavily by institutional programs at the UMBC to diversify the faculty through postdoctoral recruitment and conversion into the tenure-track. The Provost’s Fellowship for Faculty Diversity has operated for 10 years, with over 50% of the postdoctoral participants staying on as UMBC faculty. A more recent adaptation of this program, the Pre-Professoriate program at UMBC, addressed many of the ways to make this program more effective in the laboratory sciences, and the administrators of these programs are active members of the Alliance team, ensuring we incorporate lessons learned from these initiatives. Another example of the interplay between the Alliance level and Institutional level of the model is the process of finding and hiring Fellows. The recruitment and hiring of Fellows occur independently on individual campuses since funding for the fellowship comes from the institutions. But guidelines for recruitment of Fellows were developed by the Alliance leadership team for use across the Alliance institutions, and include sample job ad language, rubrics, and recruitment strategies.^2^ The Alliance team realized such guidance was necessary, since institutional processes can vary widely, and the Fellows hired are not traditional postdocs but tenure-track faculty-to-be. Using a national search process that is similar to that of a faculty search would build departmental buy-in for future conversion at the institution or another university system institution. The recruitment process has some shared components which the Alliance hopes to strengthen over time (for example, using shared recruitment venues like the annual Summer Success Institute conference for underrepresented graduate students and postdocs in STEM). New Fellows are onboarded with a combination of Alliance activities (e.g., one-on-one welcome and skills assessment^3^ with the director, Orientation with all Fellows) and institutional activities such as campus orientations, meetings with their mentors and department chairs, and the development of individual development plans (IDP) with their primary faculty mentor.

Similarly, our model’s success hinges on a reciprocal interplay between the Alliance and the USM system office (Figure 2). The Alliance encourages System change by participating in System-level committees that can influence structural changes that facilitate hiring of Fellows at institutions throughout the system, as well as influence policies to reduce bias and increase diversity (e.g., Appointment, Promotion and Tenure policy committee). In turn, the Alliance receives support from the System by being given platforms for dissemination (e.g., Academic Affairs meetings) and technological support. Most notably, at the request of the Alliance, the USM Information Technology unit is building out a database of USM postdoctoral scholars and academic opportunities (e.g., guest lectureships, adjunct teaching positions, and faculty openings) within the system. This database, modeled in part after the Big 10 Alliance’s Professorial Advancement Initiative postdoctoral directory,^4^ enhances the Alliance’s ability to connect Fellows with opportunities across the system and also vice versa, provides a mechanism for departments to learn about postdoctoral talent that already exists within the system and could bring additional diversity to their institution. We have been grateful to have incredible buy-in and support from the USM system office, and they have allowed us access to numerous system-wide meetings to describe our efforts, build relationships, and begin the process of forging policies and practices that will be critical to the success of the project.

**Figure 3:**
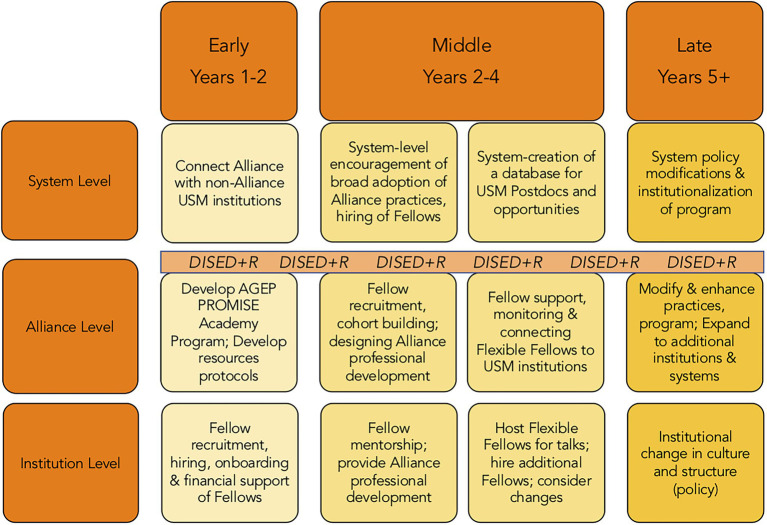
General timeline of activities of the AGEP PROMISE Academy model, separated by system (i.e., state university system office), Alliance, and institution levels.

Participation in the project is expected at all levels throughout the period of NSF funding and beyond. Some of the major activities of each level across the early, middle, and later years of the 5-year AGEP grant are captured in Figure 3. While the Alliance is committed to continuous DISED+R, the most notable development and implementation activities included creating the AGEP PROMISE Academy program and directing the execution of that program, including the cohort building, professional development, and conversion pathways to tenure-track faculty positions. The institutions recruit, hire, onboard and mentor the Fellows, but also host Fellows from other institutions for seminars and guest lectureships to consider them for faculty lines. In addition, institutions examine their own departmental “readiness” for supporting the success of additional faculty from minoritized backgrounds and consider their own institutional structural changes (more of this is discussed in Overcoming Barriers and Measuring Success, below). The USM office administrators promote the Alliance throughout the university system and facilitate engagement with non-Alliance institutions, encourage broad adoption of Alliance practices, and support infrastructure and policy changes that can institutionalize the Alliance model at the system level.

### The AGEP PROMISE Academy: Our Programmatic Core

At the center of our state system model is the postdoctoral experience in the AGEP PROMISE Academy (Figure 1). This two-year fellowship is designed to prepare the Fellow for the tenure-track, preparing them to successfully convert into a tenure-track line at their fellowship institution or at a campus within the university system.

Building a sense of community and a network across campuses is a cornerstone of our AGEP PROMISE Academy. Fellows attend monthly virtual meetings with the other Fellows and the program director to help provide an external safe space to share concerns and successes, troubleshoot barriers that arise, and to build a sense of community among these underrepresented scholars who may not have frequent access to a group of other racial minorities in a similar position. Lambert et al. (2020) recommends this type of cohort and structured programming for underrepresented minority postdoctoral scholars and cites the success of institutional efforts that leverage cohorts to find community (Eisen and Eaton, 2017). In addition to monthly meetings of the Fellows, the Fellows attend regular professional development activities led by Alliance institutions. To help guide the professional development that we offer to our Fellows, we worked with current and former Fellows (now faculty) and Alliance team members to develop a set of common learning outcomes^5^ that aligned with their experience as well as the National Postdoctoral Association’s Core Competencies^6^ that build a Fellow’s skills to enter the professoriate. We developed and implemented a skills assessment with our Fellows upon entering our program and at checkpoints during their fellowship. But mentorship of the Fellows and use of the skills assessment in an IDP occurs on the individual campuses with designated faculty mentors. A distinguishing feature of our model is that we provide a multi-institutional professional development plan that includes workshops leveraging the distinct strengths of institutions within the university system alliance. For example, the regional comprehensive institutions (TU and SU) provide pedagogical training and encourage best practices for faculty research on a predominantly undergraduate campus. Fellows then access grant-writing workshops from the medical school within our alliance (UMB) and mentorship training from the R1 institution (UMCP), while our R2 institution (UMBC) hosts underrepresented networking events and organizes Orientation and program-recruiting events. This model is effective because it draws from pre-existing programmatic elements on various campuses that simply need to be coordinated into a unified calendar. This is a culminating example of the CIS pillar “mutually reinforcing activities.”

This inter-institutional professional development has the intentional added benefit of educating the Fellows about a broad range of institutional types at which Fellows can become a faculty member. This is important because, while most postdoctoral positions are housed at research-intensive R1s, most institutions that employ tenure-track faculty are not research-intensive. Indeed, one of the common learning outcomes for the program is to expose Fellows to multiple campus environments and help them make more informed choices about where they will be most fulfilled as they establish their faculty career. Learning about the different institutions happens at structured events (such as the yearly orientation and at an annual session held at one of the regional comprehensive Alliance institutions) as well as organically through Fellow interactions with each other at monthly meetings or group sessions with AGEP PROMISE Academy Mentors in Residence (faculty from underrepresented backgrounds from around the country).

**Figure 4:**
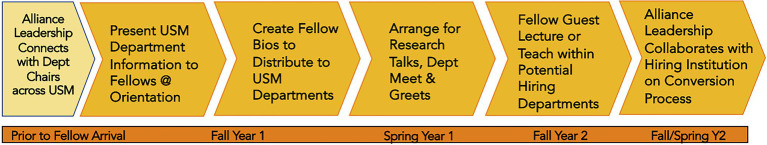
Detail of the AGEP PROMISE Academy Flexible Pathway.

Importantly, though, our program additionally focuses on *restructuring* the traditionally short-term nature of postdoctoral employment by incorporating a career pathway to a tenure-track professorship at the same institution or within the same statewide educational system where they are trained. The goal of this program is to diversify the tenure-track faculty within the university system through retention of Fellows as faculty. Alliance campuses have worked in years one-three of the project to solidify the “Predetermined pathway” (where the Fellow is retained at the campus where they are trained during the Fellowship, see Figure 1). This is based on two successful postdoc conversion programs at UMBC (Culpepper et al., 2021). Four of the nine fellows in our program are in the Predetermined pathway and four of the five campuses have a Predetermined pathway in place (the fifth campus is establishing this on their campus for a fall 2022 hire). We have simultaneously been building out a unique process we call the “Flexible pathway”—which is a greater challenge but expands the possibilities for postdocs to have a stable pathway to a tenure-track faculty position within the broader USM. The Flexible pathway model (detailed in Figure 4) requires educating Fellows and institutions about each other and facilitation of opportunities to interact in a sort of professional matchmaking process. The aforementioned database of information about Fellows and institutional opportunities, being built by the USM Information Technology team, will facilitate matchmaking. The opportunities for Fellows thus far have been research talks at universities of interest within the system, but will most likely expand to include departmental guest lectures, teaching opportunities, or full day mock faculty interviews like those offered through the Cottrell Emerging Scholars program (Diversity program helps postdocs prepare for interviews, 2020). We have two Fellows currently starting their second year of this Flexible pathway and have another beginning their first. We will be hiring additional Fellows in this pathway in the coming years, and self-study and evaluation of this pathway will certainly enhance the process.

## Overcoming Barriers and Measuring Successes

The overarching goal of this project is to develop, implement, self-study, evaluate, and disseminate a state system model to increase the number of historically underrepresented faculty in the biomedical sciences. In order to do so, we engage in multiple efforts to understand the factors that challenge and facilitate our work. Our external evaluator hosts yearly focus groups, meets with leadership, attends annual retreats and meetings and reviews documentation to help determine if we are succeeding in developing and implementing a model, and also helps identify factors that facilitate and hinder that progress. Our internal evaluator monitors institutional data to observe changes in overall faculty diversity, conducts interviews with Fellows, assesses the effectiveness of our professional development programming, and provides regular formative feedback to encourage leadership team efforts and self-study (our internal evaluator attends leadership meetings). We also have an external advisory board, made up of leaders of other institutions and organizations with experience developing and implementing programs aimed at diversifying the professoriate, and meet with them four times a year. We receive annual reports from the internal and external evaluator as well as the external advisory board each year. In addition, we have undergone two site visits by NSF program officers and external panelists in years one and three of the project to assess the strengths and weaknesses of ongoing work.

Establishing a state system model to diversify faculty comes with a number of built-in barriers to success. First, there is the simple issue of geography when dealing with institutions that are hours apart. We happen to be in a small state with most alliance institutions within 30–45 min of each other, but one of our institutions is 2.5 h away. We learned, as many have in the past year, to make good use of video conferencing and providing fellows with virtual professional development. Second, there are power dynamics at play when you build collaborations—racially, between institutions, between ranks, etc. This can manifest in a myriad of ways and can undermine collaborative decision-making. We had to be aware of these from the beginning and actively work to neutralize them when possible. Efforts like ensuring nametags had names but not titles at in-person retreats, conducting an anonymous survey about authorship determinations for dissemination, and inviting coordinator-level team members to participate actively and provide feedback has helped mitigate these dynamics. Another barrier we faced was understanding and navigating different institutional language and policy. Academia is traditionally siloed, and policies/governance is institutional, so creating a common vision and common language to use as we engage with each other across a system was very important. Within our system, different institutions have different definitions of postdoctoral scholars, for example, and not every institution has search waiver policies that could facilitate conversion of a Fellow into a faculty role. These obstacles were exposed and often at least partially surmounted by getting to know each other through retreats and group meetings, having consistent communication, as well as having accessible documentation (agendas, minutes, presentation copies, etc.) in a shared drive. For example, we decided as an Alliance to pursue system-wide search waiver language with the system office through their Appointment, Promotion and Tenure committee, hoping that the university system might adopt language that already exists on some campuses to permit the hire of a tenure-track faculty under special circumstances. This effort is ongoing, but two members of the Alliance leadership team have been appointed to the system committee reviewing the policy, which is an excellent start.

Faculty and department buy-in for initiatives will always be a requirement for institutional change that is sustainable and building buy-in or shifting culture across different departments at different institutions within a system will be an ongoing issue. This is especially true as we attempt to drive a culture shift of viewing postdoctoral fellows as future colleagues developing their own research agenda, as opposed to simply trainees gaining experience on a faculty member’s project. This paradigm shift encourages departments to support postdocs as independent researchers training to be in charge of their own labs as faculty, as well as preparing for faculty careers more broadly (e.g., teaching, engaging in service). Engaging with hiring departments, chairs, STEM Deans, and even search committees about our model has helped build nominal support, but we recognize deeper adoption will take time and positive experiences with the program/our Fellows. Currently, we are in the process of assessing what we call “departmental readiness” across potential hiring departments at alliance institutions. Using a validated, qualitative, and time-intensive instrument to interview faculty, we hope to learn about the climate of departments and their true commitments (intellectually and financially) to support the recruitment and retention of underrepresented faculty. We have plans to work on a more streamlined assessment that will help us gauge *and support* departments more effectively, helping shed light on areas for improvement and directing them to resources to assist with that improvement.

Finally, a barrier for this project is the simple truth that policy creation is slow. The Predetermined pathway for postdoc conversion has relied on working with existing institutional policies or creating policy within an institution (still may be slow, but a known approach). The Flexible pathway will require policies and practices that *cross* institutions and possibly will require conversation and approval at the level of the Board of Regents—and acceptance within each USM institution. We recognize this challenge and advise working with advocates for equity and diversity within the USM early and often as they can assist in navigating that landscape successfully.

Despite these barriers, we can report multiple successes from the project thus far. First, as has been noted by our external evaluator and NSF panelists, we have built the essential collaborative infrastructure of human resources as the CIS pillar of “backbone support”: a broadly engaged group of stakeholders, a leadership team, a program director, and key change agents at the university system office level. Focus group data from leadership and broad team members demonstrate that we have a highly functional leadership team with a project director and representatives from alliance institutions, including decision-makers, thought leaders and “doers” that implement programming directly with the Fellows. We thoughtfully constructed this leadership team with two representatives from most campuses, (1) a Dean or Vice Provost with influence over faculty or postdoctoral affairs and (2) an administrator or professional developer that engages with postdoctoral fellows and their mentors. Critically, all leadership team members and broad Alliance team members have a passion and track record of working on projects that increase diversity. To add expertise, we have curated an experienced and engaged external advisory board that provides substantive feedback that positively impacts our progress. For example, in response to feedback, we have taken on developing tools to investigate “departmental readiness” at Alliance institutions to hire, support, and advance faculty from underrepresented backgrounds. We take seriously the CIS pillar of “consistent communication” and engage in bi-weekly leadership team meetings, regular meetings with an external advisory board, and annual retreats and meetings with leadership across system universities and system office administration.

Second, we have generated a model with thoughtfully crafted programmatic elements (the AGEP PROMISE Academy, Figure 1) and a collaborative design of reciprocal influence across the Alliance, the University System of Maryland administration, and individual institutions (Figure 2). The developed model has been implemented across four of the five Alliance institutions, with the fifth institution implementing this year. We have hired nine of the 16 Fellows we set out to hire and have converted two Fellows to a tenure-track position both within the USM. Through regular self-study and integration of feedback from external and internal evaluators, this model has evolved continuously. To help us act as a unified Alliance, we have collaboratively built numerous resources for the program that are used on the campuses, such as guidelines for recruitment and hiring, onboarding checklists, common learning outcomes, mentor and mentee expectations, skills assessments, individualized development plan templates. While much of the qualitative data collection from Fellow interviews is protected and will remain confidential until the end of the grant period, we have received formative feedback that helps us see the positive impact of our program. Our Fellows have found community with each other through monthly meetings, and have engaged with cross-institution, professional development that has improved their tenure-track readiness (as has been self-assessed through a skill-assessment tool we developed). Fellows have connected to mentors and faculty outside their programs as well, especially through our mentor-in-residence program, workshops to help Fellows learn about faculty life at predominantly undergraduate institutions, and research seminars they have given at other alliance institutions. We have successfully helped expand their networks of peers, successful role models, mentors, and advocates through these activities in addition to our annual Summer Success Institute conference for underrepresented scholars in STEM.

Finally, along the way, we have created a significant culture of *systemness* and reciprocal impact: this model leverages collaboration and acknowledgment of distinct institutional strengths to create cross-institutional professional development and mentorship for our Fellows and has facilitated foundational work for novel system-wide policy. And while the goal is a system-wide approach, our work has inspired significant institutional change on alliance campuses. For example, the successful hiring and conversion of a Fellow into a tenure-track institution on Salisbury University’s campus led to the development of formalized Predetermined pathways on 3 campuses (UMCP, UMB, and TU) that had previously planned to only have Flexible Fellows. For the R1 institution, UMCP, this meant a culture and policy change as they implemented a new FAMILE initiative (Faculty Advancement at Maryland for Inclusive Learning and Excellence).^7^ In this new program, postdoctoral scholars they hire as part of the AGEP PROMISE Academy (and concurrent President’s Postdoctoral Fellowship appointment) have a pathway to a tenure-track position at the time of hire. At the professional school, UMB, the provost committed $20,000 a year toward salary for the first 2 years of postdoctoral fellowship and one additional year as a faculty member for Fellows hired into their predetermined program. And at TU, one of the regional comprehensive institutions in the Alliance, they decided to hire their first AGEP PROMISE Academy Fellow this coming academic year, as a postdoc with a pathway to conversion in the biology department. The institutional impacts extend beyond creation of conversion pathways. After hearing the research team and Alliance presentations at an annual meeting, the provost of Salisbury University invited research and leadership team members of the Alliance to come to several meetings and consult on the draft of “Plans to support Diversification and Success of Faculty.” And while UMBC’s institutional postdoc conversion program, the Provost’s Postdoctoral Fellowship for Faculty Diversity, served as the model that inspired the AGEP PROMISE Academy, that program is now benefitting from lessons we are learning at the system level: They are evaluating a departmental readiness instrument we have designed for potential use in their own program. While our model is in too early a stage to measure the impact of increasing the number of underrepresented tenure-track faculty in the biomedical sciences, early signs indicate that we are forging pathways that will yield this result.

## Discussion: Practical Implications and Lessons Learned

Though the AGEP PROMISE Academy Alliance has too few fellows to report significant outcomes thus far in terms of conversions and impacts on faculty diversity, the hypothesis that this novel model enhances faculty diversity is testable. We are confident that postdoc conversion models, particularly those that can occur on a large scale, such as within consortia or a state system, have potential to realize the academy’s hope to broaden participation and have equitable representation among faculty. The University of California’s (UC) state system model to diversify faculty, the President’s Postdoctoral Fellowship Program, started in 1984, has hired over 800 fellows, 67% of which move on to tenure-track positions, half of which are at UC institutions. Of those hired into faculty positions within the system, 98% were successful at achieving tenure and 90% have stayed within the UC system, demonstrating that state system alliances have been instrumental in increasing the number of faculty from diverse backgrounds. Programs like the UC President’s Postdoctoral Fellowship and the AGEP PROMISE Academy upend some of the known challenges associated with an academic postdoc that may discourage underrepresented scholars: funding insecurity without a path to stability (Lambert et al., 2020; Woolston, 2020a), isolation and low sense of belonging as an underrepresented minority (Yadav et al., 2020), and lack of professional support and development (Yadav et al., 2020). Interestingly, in attempts to model the academic research system, Wood et al. (2016) created simulations that suggest that job insecurity of postdocs significantly reduces their productivity, particularly as they near the end of a fixed-term contract, and advocates moving postdocs into more secure, permanent positions to improve general scientific output and return on investment. Because the postdoc-to-tenure-track transition is a known place of departure for scholars of color (Meyers et al., 2018) it is critical to address these challenges.

The AGEP PROMISE Academy employs some best practices of other postdoc conversion models, including the University of California’s model, while operating within a very different university system structure and funding model. This is important to note, because institutional, system, and state contexts will likely drive *necessary* variability between different consortia approaches. How positions are funded, system policy and language, institutional and departmental processes for hiring may look very different across an alliance. As our program is replicated with other consortia or other disciplines (currently, our AGEP PROMISE Academy focuses on the biomedical sciences), these contexts will need to be assessed and considered. The case could be made that our particular university system structure facilitated the development and implementation of this model as we have two postdoc-intensive institutions (the R1 and professional school), have multiple R2 research-intensive institutions that hire faculty with high research expectations, and have regional comprehensive institutions that, while teaching-focused, are open to hiring teaching-passionate postdocs with the hope to convert to faculty. That we have this distinct constellation of institutions with the system to comprise an Alliance may be viewed as a limitation to our ability to replicate the model in other systems, but we instead view our ability to work across these distinct institutions to successfully build a model as a sign of *increased* imitability. Most university systems have a flagship or R1 and numerous less research-intensive institutions. We have shown that all types of campuses can recruit and train postdoctoral fellows from underrepresented backgrounds, can provide meaningful professional development across a university system, and can contribute to alliance-wide protocols and practices for conversion and retention.

The multi-level collaboration with dynamic influence between the Alliance, the institutions and the university system administration, is a necessary part of the organizational change process (Kezar, 2001) that we hope to evoke at the state system level. While we have discussed numerous ways in which the implementation of this model includes changes in practice, the overall goal is to go beyond increasing diversity quantitatively and to shift culture, norms, and expectations within the system. The involvement of institution provosts, deans, department chairs, and staff, in combination with the regular engagement of key university system administrators, intentionally builds a network of responsibility for the hiring and future success of scholars from underrepresented backgrounds within the university system.

There are numerous limitations to the analysis that has been presented, including the short time frame in which this intervention has been implemented. Case studies, by nature, are descriptive investigations of a particular phenomenon with unknown generalizability. However, because this state university system model has been designed with diverse types of institutions, with leadership across divisions and ranks, and with a decentralized structure that requires each institution’s financial buy-in (and subsequently provides institutional control of how they participate in the alliance), it is likely that this model can be replicated more easily than highly centralized approaches. Finally, this article relies on data collected by self-study as well as through external sources (external evaluators, advisory board members, and NSF-appointed panelist experts), and is therefore subject to researcher bias.

Despite these limitations, the accomplishment of designing and implementing a state university system to diversify faculty is noteworthy and we feel the stage is set for success of this model, such that it can be replicated beyond the biomedical sciences and beyond the USM. With that in mind, there are several lessons learned in *building* the alliance and the model that we thought were worth mentioning. Again, these lessons have been distilled from regular self-study, review by our external advisory board, assessments and reports from our External and Internal Evaluators, and two NSF site visits.

### Leverage Existing Relationships and Seek External Expertise

External Evaluation has suggested that a positive facilitator of our success has been the history of previous collaboration among many of the institutions and institutional leaders within the alliance. Indeed, our alliance leverages relationships forged over 15 years of collaboration between three of the five institutions on a previous PROMISE AGEP that was focused on increasing enrollment and graduation of underrepresented minorities in STEM PhD programs through community building and professional development. However, not all the institutions were a part of that project, and external evaluators have noted that we have brought in the regional comprehensive institutions successfully. Intentional efforts to foster inclusion and reduce power dynamics have facilitated this according to self-study and evaluation. In focus groups with team members in year three, the external evaluator noted that team members from these institutions felt like meaningful contributors to the projects, whose expertise was respected and valued.

We also established a highly engaged external advisory board with higher education leaders and change agents who have histories of successful programmatic innovations and a passion to move the needle on faculty diversity. Our external advisory board meets with us at least four times a year: at our summer Annual Retreat, our winter Annual Meeting, and for video conference calls in the spring and fall. Finally, we took very seriously the feedback received from panelists and program officers in our two site visits organized by our funding agency, the NSF, and made meaningful shifts in the way we operated based on suggestions received.

### Commit Staff Time Meaningfully to the Project at All Alliance Institutions

In addition to having a part-time Director of the project, we established a leadership team that included two individuals from each research-intensive campus (a dean-level co-PI as well as a coordinator) and a coordinator on each teaching intensive campus: a dean or director-level co-PI. This infrastructure was not in place at the beginning of the project and was put into place upon the recommendations of our first NSF Site Visit panel in year one of the project. Since then, this group meets at least every 2 weeks to move the project forward. This structure allows for high-level knowledge and decision-making, as well as boots on the ground implementation and assessments to be communicated regularly. In addition, external evaluation has determined that engaging members of the broad team (outside of the leadership team) on subgroups that develop drafts of protocols or documents (such as the common learning outcomes) has been a practice that has benefitted the project’s progress.

Our external evaluation reports and NSF Site Visit reports (both from year one and year three) have noted that having an experienced staff leader acting as Director of the project is extremely beneficial, both for the Fellows as well as the leadership and broad project team. As part of self-study, the Director on this project tracked time spent on the project and found the work took up 35–40% of her time, while being funded for 10% of her time. The external evaluator determined that, generally, the limited time funded by the grant to run the project is a hindrance to project success. Thus, we advise that for replication efforts, institutions have a director who can dedicate (at least) 30–50% of their time to direct the project.

### Learn From Other Models to Envision the Program as Comprehensively as Possible Prior to Bringing Fellows on Board

We wish we had the time to do this more effectively, instead of a “building the plane as you fly” approach, as we have worked diligently to have success in developing and executing and assessing the model simultaneously. We have sequentially tackled standardizing learning outcomes, aligning recruitment practices, developing inter-campus professional development, and solidifying onboarding procedures over the first 3 years of the project, all while Fellows have been in place. In a review article about postdoc conversion models (Culpepper et al., 2021), authors describe five stages of program establishment and execution: (1) Laying the Foundation; (2) Recruiting Fellows, Matching to a Mentor/Department and Pre-Arrival Preparation; (3) The Fellowship Period; (4) Conversion to the Tenure-Track; and (5) Ongoing, Iterative Evaluation for Program Improvement. We highly recommend using this structure, and the resources provided within that publication, to plan out new consortia and state system models.

### Bring in Institutional and University System Leadership Early and Often

This is critical to ensure that practices and policies are taking shape that will enable project implementation. We have our Provosts, who serve as PIs on the project, and university system leadership engage with us at least twice a year at annual retreats and meetings, but frequently engage with these groups two-four additional times throughout the year in less formal settings (for example, we invited system leadership to one of our leadership team meetings, we had PIs and the broad alliance team come together to learn from an existing postdoc conversion model, etc.). These interactions and relationships lay the foundation for institutionalization of these pathways at institutional, alliance, and system levels (e.g., influencing system policy and campus initiatives).

### Have Plans for Regular Assessment

We self-study and evaluate the development of our state system model and the quality of postdoctoral experience in the program at our Annual Meetings and through internal and external evaluation. Structured self-study includes engaging the broad team of 35 senior personnel and Co-PIs across the system in staff, faculty, department chair, Dean, and Provost positions about what we are doing well and how we can improve. External evaluation includes studying the experience of the leadership team and broad Alliance team as we develop and implement the model and will include ascertaining the reflective experiences of the postdoctoral Fellows within the model at the end of their experience. Internal evaluation will help us determine the changing departmental demographics of our Alliance institutions (which we hope to influence), the impact of our professional development activities, the departmental climate and “readiness” for hiring and retaining more diverse scholars, and the ongoing experience of our Fellows. From this continuous self-study and evaluation, we have learned that several factors have likely contributed to our success: beginning with a pilot of five institutions of diverse types, ensuring regular communication across the Alliance (not just the leadership team) throughout the year, and making efforts to reduce and remove power differentials (e.g., wearing name tags with just first names and not titles). We have also made numerous changes based on this process, such as building out an Alliance Google drive and a website, providing summaries of activities to the broad team between annual retreats and meetings, adding additional mentors besides a primary research mentor, and developing an onboarding checklist for institutions bringing on a Fellow. We intend to continue to evolve our practice for our own benefit as well as the benefit of others, as we hope it facilitates our ability to assist other systems interested in replicating this unique model for diversifying faculty.

## Conclusion

Here, we report significant progress in the development and implementation of a novel state university system approach for diversifying faculty in the biomedical sciences. While these efforts are still ongoing, this is an important case study from which to monitor and learn. The Alliance has only just entered year four and looks forward to providing more comprehensive analysis in future reports, including the perspective of the fellows, structural and climate changes occurring on participating campuses, and impacts to the diversity of biomedical faculty at Alliance institutions. As postdoc conversion models for underrepresented minority scholars are growing at a number of institutions, it is our sincere hope that state universities will consider a collaborative model like ours to expand the power and success of those programs beyond their individual institutions and that the lessons we have learned in overcoming barriers and finding success will facilitate adoption and adaptation of similar models in other state university systems. It is imperative that we work together to address the underrepresentation of minority scholars within faculty ranks. We encourage campuses to engage with their system office leadership and find advocates that will be genuine partners on these projects; to build multi-level commitments from institutional, college, and departmental leadership; and to be open to working through the inherent challenges of working with different types of institutions across a broad geographic area.

## Data Availability Statement

The original contributions presented in the study are included in the article/supplementary material, further inquiries can be directed to the corresponding author.

## Author Contributions

RC, CG, JR, WC-V, JA, JB, BE, EG, YM, and MS collaborated heavily on the development, implementation, and self-study of the model described in the paper, and all contributed intellectually to the paper. RC was the manager of the project and did most of the writing. CG created the outline of the paper, determined basic content, and helped review the literature and collect citable resources. CG drafted the introduction and context sections while RC drafted the section about the model and the discussion. BE, EG, JA, JB, JR, MS, YM, and WC-V added comments, suggestions, and edits at multiple stages. All authors contributed to the article and approved the submitted version.

## Funding

The AGEP PROMISE Academy Alliance is supported by the National Science Foundation (NSF), Directorate for Education and Human Resources (EHR), Division of Human Resource Development (HRD), Alliances for Graduate Education and the Professoriate (AGEP) Awards: University of Maryland Baltimore County (UMBC; 1820984), University of Maryland College Park (UMCP; 1820975), University of Maryland at Baltimore (UMB; 1820983), and Salisbury University (SU; 1820971), and Towson University (TU; 1820974). The award to UMBC (1820984) is being used for author fees for this publication.

## Conflict of Interest

The authors declare that the research was conducted in the absence of any commercial or financial relationships that could be construed as a potential conflict of interest.

## Publisher’s Note

All claims expressed in this article are solely those of the authors and do not necessarily represent those of their affiliated organizations, or those of the publisher, the editors and the reviewers. Any product that may be evaluated in this article, or claim that may be made by its manufacturer, is not guaranteed or endorsed by the publisher.

## Footnotes

^1^The NSF defines underrepresented minorities in STEM as African Americans, Hispanic Americans, American Indians, Alaska Natives, Native Hawaiians, and Native Pacific Islanders.

^2^ https://theageppromiseacademy.com/wp-content/uploads/2021/10/Guidance-for-Recruitment-and-Selection-of-APAA-Fellows.pdf

^3^ https://theageppromiseacademy.com/wp-content/uploads/2021/10/APAA-CLO-SKills-Assessment-Revised.pdf

^4^ https://btaa-pai.btaa.org/pai

^5^ https://theageppromiseacademy.com/wp-content/uploads/2021/10/APAA-CLOS-and-Expectations_20210803.pdf

^6^ https://www.nationalpostdoc.org/page/CoreCompetencies

^7^ https://www.faculty.umd.edu/media/183/download
